# Disseminated Histoplasmosis in Very Early Diagnosed De Novo STAT3-HIES

**DOI:** 10.1007/s10875-025-01923-w

**Published:** 2025-10-17

**Authors:** Armen Sanosyan, Ran Hazan, Alexandra F. Freeman, Erica G. Schmitt, Caroline C. Horner

**Affiliations:** 1 Department of Pediatrics, Division of Allergy and Pulmonary Medicine, Washington University in St. Louis, St. Louis, MO USA; 2 Department of Medicine, Division of Allergy and Immunology, Washington University in St. Louis, St. Louis, MO USA; 3 Department of Pediatrics, Division of Rheumatology and Immunology, Washington University in St. Louis, St. Louis, MO USA; 4 Laboratory of Clinical Immunology and Microbiology, National Institute of Allergy and Infectious Diseases, National Institutes of Health, Bethesda, MD USA

**Keywords:** STAT3-HIES, *Histoplasma capsulatum*, Appendicitis

## To the Editor

Hyper-IgE syndrome caused by dominant-negative STAT3 pathogenic variants (STAT3-HIES) is a rare inborn error of immunity (IEI) characterized by extremely elevated IgE levels, recurrent pneumonia, staphylococcal skin abscesses, and non-immunologic manifestations, such as skeletal and vascular abnormalities. Although not part of the characteristic triad of symptoms, localized and systemic fungal infections often prove deleterious in STAT3-HIES due to impaired Th17 function. The mean age of diagnosis for de novo forms of STAT3-HIES in the USA is the second decade of life, whereas invasive fungal infections are more commonly seen in the fourth decade [1].

Here, we describe a case of early-life diagnosis of de novo STAT3-HIES, further complicated by disseminated histoplasmosis causing appendicitis. To our knowledge, this is the earliest documented case of de novo STAT3-HIES and the first report of histoplasma-associated appendicitis in this disease.

**Fig. 1:**
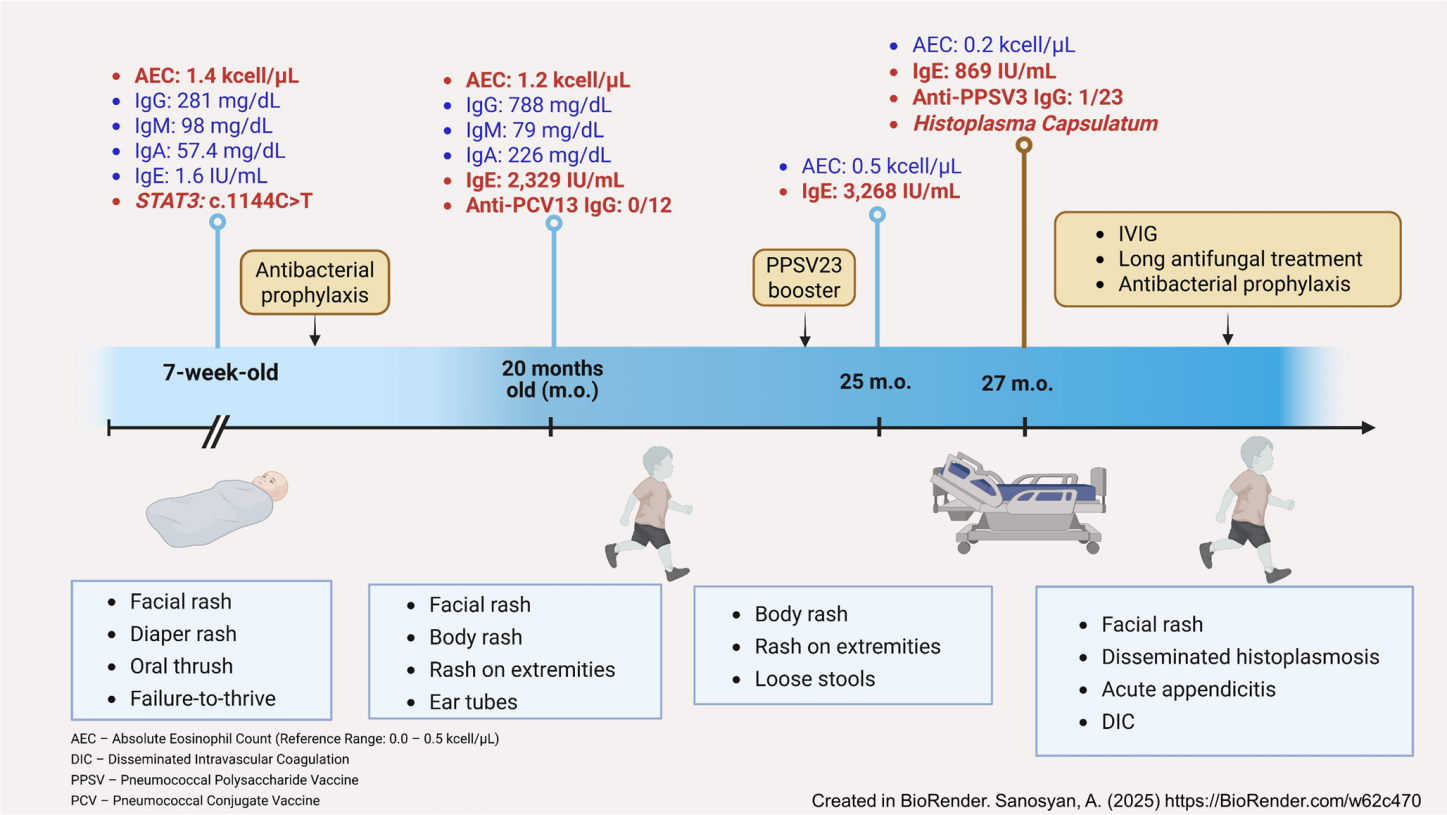
This is mandatory. Please provide

A 7-week-old Caucasian male, born from a full-term pregnancy and living with his family in rural Illinois, was admitted to the inpatient unit with diarrhea, vomiting, failure to thrive, a facial maculopapular rash, shallow perianal ulcerations, and oral thrush unresponsive to nystatin treatment. The complete blood count revealed eosinophilia at 1,400 cells/µL. His newborn screen for T-cell receptor excision circles was normal, as were his T, B, and NK cell counts, immunoglobulin profile including IgE level, and neutrophil oxidative burst (Fig. 1). A genetic panel for IEI identified a *STAT3* heterozygous pathogenic variant, c.1144 C > T (p.Arg382Trp), absent in both parents.

His dehydration, diarrhea and vomiting were managed by switching to an elemental formula, and his skin rash responded to topical hydrocortisone. The oral thrush resolved with fluconazole, and the patient was discharged on trimethoprim-sulfamethoxazole prophylaxis, eczema care, and scheduled periodic follow-ups.

By 20 months of age, he had experienced three episodes of otitis media requiring ear tube placement, occasional eczema flares, and loose stools. His eosinophil count remained elevated at 1,200 cells/µL, and his IgE level was elevated for the first time to 2,329 IU/mL. Although his total IgG was normal, his response to the pneumococcal conjugate vaccine and subsequent PPSV23 booster were inadequate. His loose stools were initially attributed to lactose intolerance, with mild improvement on a lactose-free diet.

At 27 months of age, the patient presented to the emergency department after two months of diarrhea, vomiting, and gradual refusal to walk. On presentation, he was febrile and ill-appearing, with abdominal pain, significant pallor, and irritability. Physical examination revealed discrete annular eroded papules on his facial skin and vertex, along with hemorrhagic crusts under his fingernails. Laboratory evaluation showed anemia (hemoglobin: 7.0 g/dL), thrombocytopenia (platelets: 49 kcell/µL), elevated neutrophils (absolute neutrophil count: 13.8 kcell/µL), elevated inflammatory markers (CRP: 34.8 mg/L), and signs of disseminated intravascular coagulation. Chest X-ray showed hazy perihilar and right upper lobe opacities, with small lung volumes likely indicating atelectasis. An abdominal CT scan was suggestive of appendicitis (Supplemental data).

He was initially treated with broad-spectrum antibiotics and amphotericin B. Due to a lack of improvement, he underwent an appendectomy on the third day of admission for source control.

Blood cultures were negative for bacterial growth, but cell-free *Histoplasma capsulatum* DNA was detected via the Karius test (Karius, Redwood City, CA), and urine testing was positive for *Histoplasma/Blastomyces* antigen via enzyme immunoassay. Subsequent biopsies from the scalp lesions, and the inflamed appendix tissue were positively stained for *Histoplasma capsulatum* (Supplemental Data).

Following the appendectomy, the patient’s condition improved gradually, and he was placed on prolonged antifungal therapy with itraconazole. His post-discharge course, while immunologically stable, was complicated by a bilateral femoral fracture after a fall. At the time of preparing this letter, the patient is recovering from the fracture and continues receiving IVIG, antibacterial and antifungal prophylaxis, and vitamin D.

This case represents a rare entity of early-onset, multifaceted manifestations of de novo STAT3-HIES, characterized by both immunological and non-immunological features.

Initially described before the discovery of IgE, the syndrome was named “Job’s syndrome,” referencing the characteristic skin abscesses that lack significant signs of inflammation [1]. Advances in immunology later revealed that dominant negative variants in the *STAT3* gene are the major cause of autosomal dominant HIES. This variant disrupts signaling through IL-6, IL-10, IL-11, IL-21, and IL-23. Although B cells in STAT3-HIES can mount a T-cell-dependent response, they exhibit decreased somatic hypermutation in IgE, IgG1, IgG3, and IgA1 transcripts. This, along with impaired mast cell degranulation, may explain the lower allergy rates in STAT3-HIES despite markedly elevated total IgE [1, 2].

Skin manifestations are often the earliest presentation of the disease but are frequently misclassified as “newborn rash” or “eczema.” Signs of immune deficiency progress insidiously, with 60% of cases experiencing at least one ear or sinus infection by 30 months of age [3]. The median age of diagnosis often occurs in the second decade of life, after severe infections and non-immunological complications prompt investigation into a primary immune or genetic etiology. In this case, resistant rash, mucosal thrush, and elevated eosinophil counts led to early genetic testing, confirming the diagnosis by nine weeks of age when IgE levels were still normal. Notably, infectious complications developed alongside the rise in IgE levels.

This case is unique for its disseminated histoplasmosis and histoplasma-associated appendicitis.

A case series by Odio et al. [4] detailed the natural course of endemic fungal infections in STAT3-HIES, emphasizing their frequent involvement of the gastrointestinal tract. *Histoplasma capsulatum* is an endemic fungus, typically inhaled as aerosolized microconidia, and causes pulmonary disease in a minority of exposed individuals. Endemic areas include the Ohio and Mississippi River valleys, Central and South America, parts of Africa, and regions of Asia such as India, China, and Southeast Asia [5]. In immunocompromised patients, it can disseminate, often affecting the gastrointestinal tract, particularly the colon and ileum, due to abundant lymphoid tissue. Previous cases in STAT3-HIES patients predominantly presented with abdominal symptoms, occasionally mimicking Crohn’s disease [4].

*Histoplasma capsulatum* as a cause of appendicitis and mesenteric adenitis has been reported in both immunocompetent and immunocompromised patients. In immunocompromised individuals, the unusual gastrointestinal (GI) manifestations of histoplasmosis often include obstructive luminal masses and well-formed granulomas with a higher fungal burden. Other GI presentations may involve ulcers, hemorrhagic lesions, or even normal mucosa. Histological findings frequently include diffuse lymphohistiocytic infiltration, ulceration, lymphohistiocytic nodules, and, rarely, well-formed granulomas [6].

Since STAT3-HIES patients exhibit impaired Th17 cell differentiation, these cells may play an essential role in GI tract host defense against *Histoplasma*.

In the described case, prominent gastrointestinal and skin findings likely resulted from dissemination following a subclinical pulmonary infection. Once endemic fungal infections are detected in these patients, lifelong antifungal prophylaxis is recommended.

Although early reports on hematopoietic stem cell transplantation (HSCT) in HIES were not encouraging, recent data from small series have shown favorable outcomes in terms of skin and lung infections, and pulmonary function. Improvements in IgE levels and Th17 cell counts have also been reported. However, non-immunological manifestations, such as vasculopathies and connective tissue complications likely persist [1].

The decision to pursue HSCT should involve a careful, weighted discussion that considers the factors mentioned above, the disease’s progression, and the lack of definitive treatment options for STAT3-HIES.

## Supplementary Information

Below is the link to the electronic supplementary material.

Supplementary figure 1 (PNG 754 KB)

High Resolution Image (TIF 4.29 MB)

## Data Availability

No datasets were generated or analysed during the current study.
