# Supplementary figures and images for: Disseminated Histoplasmosis in Very Early Diagnosed De Novo STAT3-HIES
Source: J Clin Immunol. 2025 Oct 17;45(1):145. doi: 10.1007/s10875-025-01923-w (PMC12534248; doi:10.1007/s10875-025-01923-w)

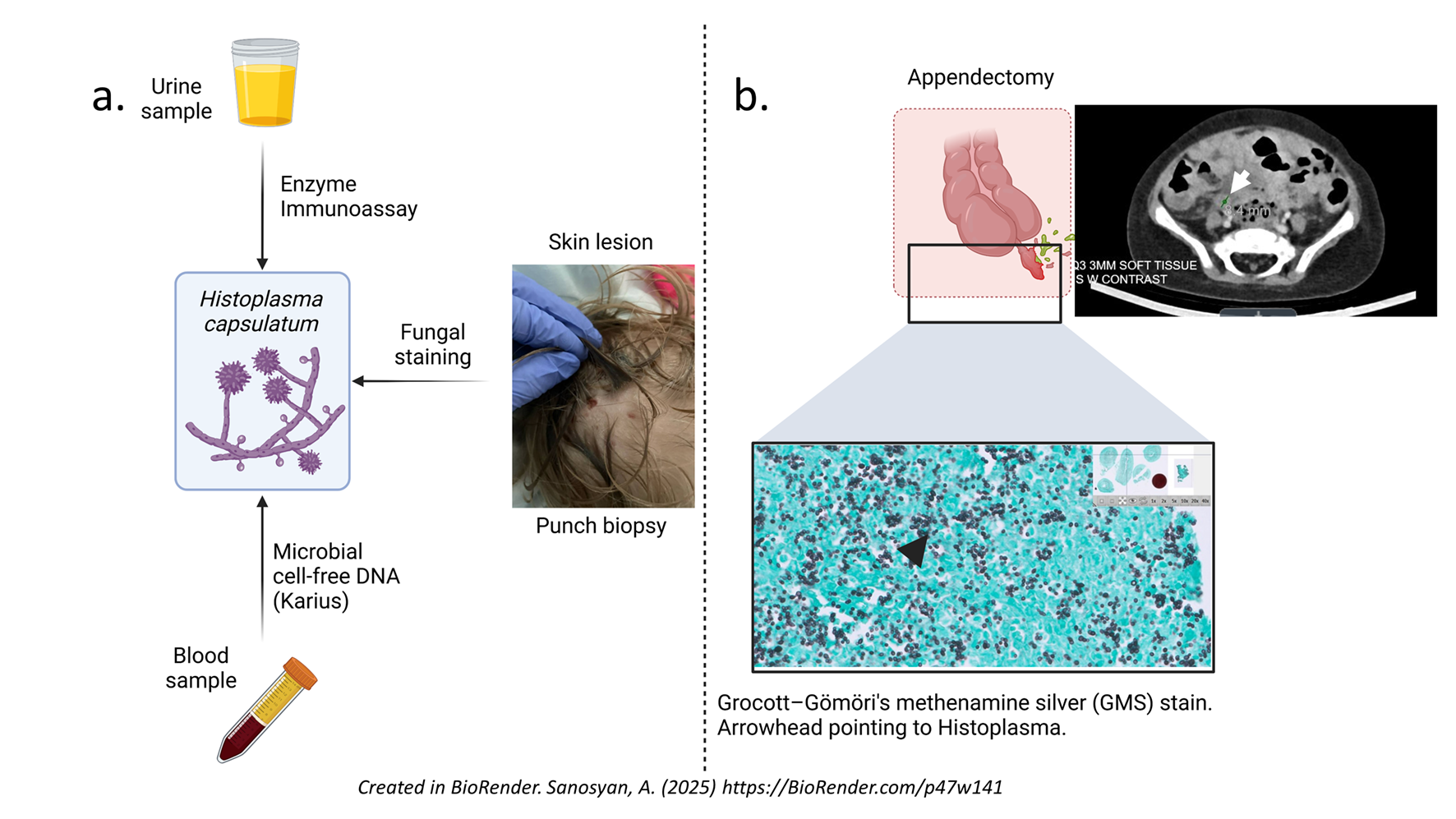

Supplement: Supplementary file 1 — (PNG 754 KB) [file 10875_2025_1923_Fig2_ESM.png]

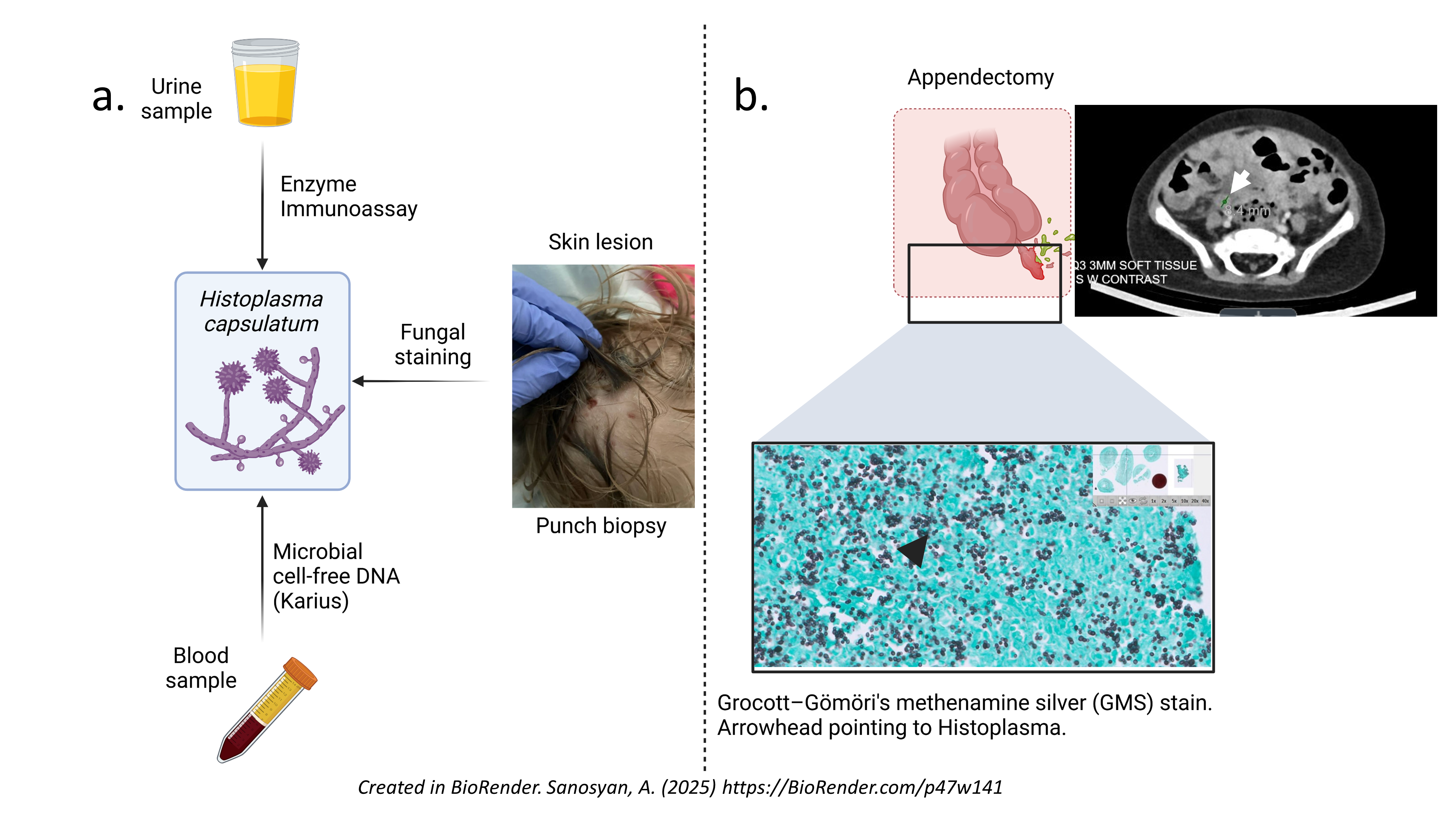

Supplement: Supplementary file 2 — High Resolution Image (TIF 4.29 MB) [file 10875_2025_1923_MOESM1_ESM.tif]
